# Supplementary material for: Picks in the Fabric of a Polyploidy Complex: Integrative Species Delimitation in the Tetraploid Leucanthemum Mill. (Compositae, Anthemideae) Representatives
Source: Biology (Basel). 2023 Feb 10;12(2):288. doi: 10.3390/biology12020288 (PMC9953438; doi:10.3390/biology12020288)
Supplement: Supplementary file 1 [file biology-12-00288-s001.zip › ES04.pdf]

Table ES04. List of samples used for the ddRAD analysis with information on voucher specimens in the Botanical Museum Berlin (B) and ploidy, collection localities, coordinates, and collectors.

Sample	Taxon	Ploidy	Voucher specimens	Locality	Coordinates (latitude, longitude)	Collectors
**135-05**	*L. ageratifolium* Pau	2*x*	B100386712	FR, Occitania, Pyrénées-Orientales, La Vallée Heureuse, 410 m	42.5038, 2.9603	*Konowalik KK42 & Ogrodowczyk*
**M60-01**	*L. ageratifolium* Pau	2*x*	B100345012, B100345013	ES, Castile-La Mancha, Cuenca, Salinas de Manzano, 1157 m	40.1019, -1.521	*Cordel 60*
**M60-011**	*L. ageratifolium* Pau	2x	B100345012, B100345013	ES, Castile-La Mancha, Cuenca, Salinas de Manzano, 1157 m	40.1019, -1.521	*Cordel 60*
**90-01**	*L. burnatii* Briq. & Cavill.	2x	B100464678	FR, Provence-Alpes-Côte d'Azur, Alpes-Maritimes, Plateau de Calern, 1235 m	43.7607, 6.9165	*Vogt 16615 et al.*
**92-02**	*L. burnatii* Briq. & Cavill.	2x	B100464676, B100464675	FR, Provence-Alpes-Côte d'Azur, Bouches-du-Rhône, Col des Portes, 650 m	43.5450, 5.6626	*Vogt 16618 et al.*
**L1008**	*L. eliasii* (Sennen & Pau) Sennen & Pau	2x	B100484003	ES, Castile and León, Burgos, Rio Ubierna, 880 m	42.5030, -3.7060	*Lopéz 2537 & al.*
**L1009**	*L. eliasii* (Sennen & Pau) Sennen & Pau	2x	B100484004	ES, Castile and León, Burgos, San Martín de Ubierna, 920 m	42.5070, -3.7050	*Galán Cela 576 & Martín*
**209-01**	*L. gaudinii* Dalla Torre	2x	B100386664	CH, Bern, Interlaken-Oberhaslit, Kleine Scheidegg, 2260 m	46.5781, 7.9700	*Tomasello TS88*
**270-01**	*L. gaudinii* Dalla Torre	2x	B100413007	AT, Carinthia, Spittal an der Drau, Torscharte, 2200 m	47.0025, 13.5275	*Oberprieler 10859*
**276-01**	*L. gaudinii* Dalla Torre	2x	B100413015	AT, Carinthia, Feldkirchen, Falkert, 2270 m	46.8603, 13.8172	*Oberprieler 10866*
**451-01**	*L. gaudinii* Dalla Torre	2x	No voucher	PL, Lesser Poland, Giewont, 1860 m	49.2505, 19.9343	*Konowalik 20160909-01*
**84-10**	*L. gracilicaule* (Dufour) Pau	2x	B100386704	ES, Valencian Community, Alicante, Benirama, 296 m	38.8379, -0.1853	*Konowalik KK20 & Ogrodowczyk*
**85-04**	*L. gracilicaule* (Dufour) Pau	2x	B100386702	ES, Valencian Community, Valencia, Altury, 337 m	39.3135, -0.6810	*Konowalik KK25 & Ogrodowczyk*
**116-01**	*L. graminifolium* (L.) Lam.	2x	B100464684, B100464683	FR, Occitania, Hérault, Col du Perthus, 802 m	43.7761, 3.2386	*Vogt 16693 & al.*

Sample	Taxon	Ploidy	Voucher specimens	Locality	Coordinates (latitude, longitude)	Collectors
**96-03**	*L. graminifolium* (L.) Lam.	2x	B100464663	FR, Occitania, Aude, Roc de L´Aigle, 560-600 m	43.1494, 2.6294	*Vogt 16656 & al.*
**162-03**	*L. halleri* (Vitman) Ducommun	2x	B100386798	DE, Bavaria, Garmisch-Partenkirchen, Meilerhütte, 2345 m	47.4134, 11.1277	*Konowalik KK67 & Tomasello*
**162-031**	*L. halleri* (Vitman) Ducommun	2x	B100386798	DE, Bavaria, Garmisch-Partenkirchen, Meilerhütte, 2345 m	47.4134, 11.1277	*Konowalik KK67 & Tomasello*
**208-01**	*L. halleri* (Vitman) Ducommun	2x	B100386672	FR, Valais, Sion, Col du Sanetsch, 2320 m	46.3308, 7.2911	*Tomasello TS65*
**280-01**	*L. laciniatum* Huter & al.	2x	B100464203	IT, Calabria, Cosenza, Col del Dragone, 1580 m	39.9020, 16.1144	*Tomasello 420*
**280-02**	*L. laciniatum* Huter & al.	2x	B100464203	IT, Calabria, Cosenza, Col del Dragone, 1580 m	39.9020, 16.1144	*Tomasello 420*
**366-01**	*L. legraeanum* (Rouy) B.Bock & J.-M.Tison	2x	B100486634, B100486635, B100486636, B100486637, B100486638	FR, Provence-Alpes-Cote d'Azur, Massif des Maures, 410 m	43.1986, 6.3151	*Vogt 17189*
**369-01**	*L. legraeanum* (Rouy) B.Bock & J.-M.Tison	2x	B100486648, B100486649	FR, Provence-Alpes-Cote d'Azur, Massif des Maures, Collabrières, 210 m	43.2444, 6.3377	*Vogt 17192*
**384-01**	*L. legraeanum* (Rouy) B.Bock & J.-M.Tison	2x	B100627809, B100627810	FR, Provence-Alpes-Cote d'Azur, Massif des Maures, 410 m	43.1988, 6.3151	*Vogt 17434 & al.*
**406-01**	*L. ligusticum* Marchetti & al.	2x	B100627838, B100627839	IT, Liguria, La Spezia, Rochetta di Vara, 210 m	44.2470, 9.7728	*Vogt 17460 & al.*
**412-01**	*L. ligusticum* Marchetti & al.	2x	B100627849, B100627850, B100627851	IT, Liguria, Genova, Valetti di Varese, 700 m	44.3603, 9.5105	*Vogt 17468 & al.*
**416-01**	*L. ligusticum* Marchetti & al.	2x	B100627855, B100627856	IT, Liguria, Genova, Piandifieno, 250 m	44.3458, 9.4588	*Vogt 17471 & al.*
**273-02**	*L. lithopolitanicum* (E.Mayer) Polatschek	2x	B100413012	SL, Central Slovenia, Kaminško Savinjske Alpe, 2100 m	46.3633, 14.5715	*Oberprieler 10862*
**274-02**	*L. lithopolitanicum* (E.Mayer) Polatschek	2x	B100413013	AT, Carinthia, Völkermarkt, Vellacher Kotschna, 1999 m	46.375, 14.5663	*Oberprieler 10864*
**128-01**	*L. monspeliense* (L.) H.J.Coste	2x	B100464618	FR, Occitania, Garde, Mas Méjean, 750 m	44.0888, 3.5786	*Vogt 16712 & al.*

Sample	Taxon	Ploidy	Voucher specimens	Locality	Coordinates (latitude, longitude)	Collectors
**131-02**	*L. monspeliense* (L.) H.J.Coste	2x	B100464615	FR, Occitania, Garde, Gardon de St. Jean, 380 m	44.1412, 3.7316	*Vogt 16716 & al.*
**340-01**	*L. monspeliense* (L.) H.J.Coste	2x	B100486666, B100486667	FR, Occitania, Aveyron, Decanzeville, 184 m	44.5822, 2.1840	*Vogt 17156 & al.*
**40-09**	*L. pluriflorum* Pau subsp. *pluriflorum*	2x	B100413758	ES, Galicia, Coruña, Cabo Finisterre, 100 m	42.8838, -9.2726	*Hößl 40*
**42-04**	*L. pluriflorum* Pau subsp. *pluriflorum*	2x	No voucher	ES, Galicia, Coruña, Caión – Lemaio, 150 m	43.3069, -8.6186	*Hößl 42*
**55-01**	*L. pluriflorum* Pau subsp. *pluriflorum*	2x	B100413749	ES, Galicia, Lugo, Cangas, 10 m	43.6309, -7.3330	*Hößl 55*
**60-01**	*L. pluriflorum* subsp. *cantabricum* (Font Quer & Guinea) T.Ott & al.	2x	B100413746	ES, Galicia, Os Ancares, Piornedo, 1530 m	42.8315, -6.8569	*Hößl 60*
**62-01**	*L. pluriflorum* subsp. *cantabricum* (Font Quer & Guinea) T.Ott & al.	2x	B100413744	ES, Galicia, Lugo, Murias, 750 m	42.9249, -6.8657	*Hößl 62*
**159-11**	*L. pluriflorum* subsp. *gallaecicum* (Rodr.Oubiña & S.Ortiz) T.Ott & al.	2x	B100386789, B100420775, B100464989	ES, Galicia, Pontevedra, Basadre, 375 m	42.8498, -7.9878	*Konowalik KK67 & Ogrodowczyk*
**161-03**	*L. pluriflorum* subsp. *gallaecicum* (Rodr.Oubiña & S.Ortiz) T.Ott & al.	2x	No voucher	ES, Galicia, Corunna, Barazon Grande, 380 m	42.8533, -7.9994	*Konowalik s.n. & al.*
**446-01**	*L. rotundifolium* (Willd.) DC.	2x	No voucher	PL, Podkarpackie, Bieszczady, 920 m	49.1191, 22.5776	*Konowalik 20180622-02-01*
**447-01**	*L. rotundifolium* (Willd.) DC.	2x	No voucher	RO, Bihor, Bihor, 1230 m	46.5189, 22.6613	*Konowalik 20180713-03-01*
**448-01**	*L. rotundifolium* (Willd.) DC.	2x	No voucher	RO, Hunedoara, Râu de Morit, 1140 m	45.3159, 22.7705	*Konowalik 20180807-03-01*
**449-01**	*L. rotundifolium* (Willd.) DC.	2x	No voucher	BH, Central Bosnia Canton, Vranica Planina, 1860 m	43.95782, 17.7403	*Konowalik 20180714-03-01*

Sample	Taxon	Ploidy	Voucher specimens	Locality	Coordinates (latitude, longitude)	Collectors
**450-01**	*L. rotundifolium* (Willd.) DC.	2x	No voucher	PL, Lesser Poland Voivodeship, Sucha County, Babia Góra, 1100 m	49.5879, 19.5515	*Konowalik 20170920-01*
**278-01**	*L. tridactylites* (A.Kern. & Huter) Huter & al.	2x	B100464207	IT, Abruzzo, Pescara, Majella, Blockhaus, 2080 m	42.1384, 14.1101	*Tomasello 417*
**278-05**	*L. tridactylites* (A.Kern. & Huter) Huter & al.	2x	B100464207	IT, Abruzzo, Pescara, Majella, Blockhaus, 2080 m	42.1384, 14.1101	*Tomasello 417*
**225-02**	*L. virgatum* (Desr.) Clos	2x	B100411746	FR, Provence-Alpes-Côte d'Azur, Alpes-Maritimes, Lantosque – St.-Jean-la-Rivière, 430 m	43.9538, 7.2961	*Vogt 16892 & Oberprieler*
**250-01**	*L. virgatum* (Desr.) Clos	2x	B100350169, B100350172	IT, Liguria, Savona, Ranzo – Ortovero, 215 m	44.0596, 8.0583	*Vogt 16932 & Oberprieler*
**184-01**	*L. vulgare* Lam. subsp. *vulgare*	2x	B100346626	BH, Gacko, Nevesinje, Ribari – Rilja, 930 m	43.2403, 18.3364	*Vogt 16806 & Prem-Vogt*
**58-02**	*L. vulgare* Lam. subsp. *vulgare*	2x	B100413748	ES, Galicia, Lugo, Santa Mariña – Reboredo, 490 m	42.8205, -7.9504	*Hößl 58*
**94-01**	*L. vulgare* Lam. subsp. *vulgare*	2x	B100464674	FR, Occitania, Aude, Carcassone, Montlaur, 160 m	43.1294, 2.6073	*Vogt 16641 & al.*
**L046-07**	*L. vulgare* Lam. subsp. *vulgare*	2x	B100550249	DE, Bavaria, Regensburg, Deuerling, 450 m	49.0333, 11.8833	*Eder & Oberprieler s.n.*
**M02-01**	*L. vulgare* subsp. *barrelieri* (Dufour ex DC.) O.Bolós & Vigo (=*L. pyrenaicum* Vogt & al.)	2x	B100297950	ES, Aragón, Huesca, San Juan de la Peña, 755 m	42.5250, -0.6690	*Cordel 2*
**266-01**	*L. vulgare* subsp. *barrelieri* (Dufour ex DC.) O.Bolós & Vigo (=*L. pyrenaicum* Vogt & al.)	2x	B100464208	ES, Aragon, Huesca, Balneario de Panticosa, 1650 m	42.7806, -0.2467	*Tomasello TS382*
**266-02**	*L. vulgare* subsp. *barrelieri* (Dufour ex DC.) O.Bolós & Vigo (=*L. pyrenaicum* Vogt & al.)	2x	B100464208	ES, Aragon, Huesca, Balneario de Panticosa, 1650 m	42.7806, -0.2467	*Tomasello TS382*
**267-03**	*L. vulgare* subsp. *barrelieri* (Dufour ex DC.) O.Bolós & Vigo (=*L. pyrenaicum* Vogt & al.)	2x	B100464210	ES, Aragon, Huesca, Angel Orus el Forcau, 2000 m	42.6327, 0.4530	*Tomasello TS392*
**45-01**	*L. cantabricum* Sennen	4x	B100413756	ES, Galicia, O Eume, Portocobo – Aurela, 350 m	43.3972, -8.0959	*Hößl 45*

Sample	Taxon	Ploidy	Voucher specimens	Locality	Coordinates (latitude, longitude)	Collectors
**50-01**	*L. cantabricum* Sennen	4x	B100413752	ES, Galicia, Ortegal, Viñan – Serantes, 10 m	43.6749, -7.9022	*Hößl 50*
**50-02**	*L. cantabricum* Sennen	4x	B100413752	ES, Galicia, Ortegal, Viñan – Serantes, 10 m	43.6749, -7.9022	*Hößl 50*
**50-03**	*L. cantabricum* Sennen	4x	B100413752	ES, Galicia, Ortegal, Viñan – Serantes, 10 m	43.6749, -7.9022	*Hößl 50*
**66-01**	*L. crassifolium* (Lange) Lange	4x	B100413740	ES, Asturias, Luanco, Cabo de Peñas, 60 m	43.6565, -5.8510	*Hößl 66 & Himmelreich*
**66-02**	*L. crassifolium* (Lange) Lange	4x	B100413740	ES, Asturias, Luanco, Cabo de Peñas, 60 m	43.6565, -5.8510	*Hößl 66 & Himmelreich*
**69-01**	*L. crassifolium* (Lange) Lange	4x	B100413737	ES, Cantabria, San Vincente de la Barquera, 50 m	43.3943, -4.3571	*Hößl 69 & Himmelreich*
**69-02**	*L. crassifolium* (Lange) Lange	4x	B100413737	ES, Cantabria, San Vincente de la Barquera, 50 m	43.3943, -4.3571	*Hößl 69 & Himmelreich*
**424-01**	*L. delarbrei* Timb.-Lagr. subsp. *delarbrei*	4x	B100605498, B100605497, B100605489	FR, Auvergne-Rhone-Alpes, Cantal, l'Arpon du Diable, 1688 m	45.0569, 2.7461	*Vogt 17487 & Prem-Vogt*
**425-01**	*L. delarbrei* Timb.-Lagr. subsp. *delarbrei*	4x	B100605488, B100605487, B100605486, B100605501	FR, Auvergne-Rhône-Alpes, Cantal, Col d´Eylac – Brèche de Rolland, 1553 m	45.1050, 2.6847	*Vogt 17489 & Prem-Vogt*
**427-01**	*L. delarbrei* Timb.-Lagr. subsp. *delarbrei*	4x	B100605494	FR, Auvergne-Rhone-Alpes, Puy-de-Dôme, Puy de Sancy, 1818 m	45.5286, 2.8128	*Vogt 17494 & Prem-Vogt*
**428-01**	*L. delarbrei* Timb.-Lagr. subsp. *delarbrei*	4x	B100605492, B100603419	FR, Auvergne-Rhone-Alpes, Puy-de-Dôme, Puy de Sancy, 1457 m	45.5364, 2.8125	*Vogt 17495 & Prem-Vogt*
**101-01**	*L. delarbrei* subsp. *ruscinonense* (Jean. & Timb.-Lagr.) Vogt & al.	4x	B100464658, B100464657	FR, Occitania, Aude, Conques-sur-Orbiel – Lastours, 193 m	43.3279, 2.3835	*Vogt 16670 & al.*
**110-01**	*L. delarbrei* subsp. *ruscinonense* (Jean. & Timb.-Lagr.) Vogt & al.	4x	B100464637	FR, Occitania, Hérault, Olargues – Bédarieux, 214 m	43.5786, 3.0212	*Vogt 16685 & al.*
**139-01**	*L. delarbrei* subsp. *ruscinonense* (Jean. & Timb.-Lagr.) Vogt & al.	4x	B100386786	ES, Catalonia, Girona, Maçanet de Cabrenys, 1015 m	42.412, 2.7504	*Konowalik KK46 & Ogrodowczyk*
**349-01**	*L. delarbrei* subsp. *ruscinonense* (Jean. & Timb.-Lagr.) Vogt & al.	4x	B100486690, B100486691, B100486692, B100486693	FR, Occitania, Tarn, Brassac, Rochers de Sarrasy, 502 m	43.6236, 2.4984	*Vogt 17171 & al.*

Sample	Taxon	Ploidy	Voucher specimens	Locality	Coordinates (latitude, longitude)	Collectors
**355-03**	*L. delarbrei* subsp. *ruscinonense* (Jean. & Timb.-Lagr.) Vogt & al.	4x	B100486699, B100486700	FR, Occitania, Hérault, Lodève, Roure de Millau, 182 m	43.7384, 3.3283	*Vogt 17177 & al.*
**106-01**	*L. ircutianum* DC. subsp. *ircutianum*	4x	B100464641	FR, Occitania, Tarn, Mazamet, 410 m	43.4816, 2.3717	*Vogt 16678 & al.*
**177-01**	*L. ircutianum* DC. subsp. *ircutianum*	4x	B100346630	ME, Cetinje, Lovćen, Bieloši, 920 m	42.3681, 18.8903	*Vogt 16794 & Prem-Vogt*
**343-01**	*L. ircutianum* DC. subsp. *ircutianum*	4x	B100486672, B100486673	FR, Occitania, Aveyron, Decanzeville, Firmi, 470 m	44.5589, 2.3114	*Vogt 17159 & al.*
**437-01**	*L. ircutianum* DC. subsp. *ircutianum*	4x	B101003356	FR, Corsica, Corse-du-Sud, Ghisoni – Cozzano, 920 m	41.9727, 9.1843	*Vogt 17865 & al.*
**87-01**	*L. ircutianum* DC. subsp. *ircutianum*	4x	B100464680	IT, Piemont, Cuneo, Roccavione, 670 m	44.2982, 7.5052	*Vogt 16611 & al.*
**L052-02**	*L. ircutianum* DC. subsp. *ircutianum*	4x	No voucher	DE, Bavaria, Regensburg, 385 m	48.9833, 12.0833	*Eder s.n.*
**L055-03**	*L. ircutianum* DC. subsp. *ircutianum*	4x	B100627297	DE, Bavaria, Garmisch-Partenkirchen, Laber, 1430 m	47.5833, 11.1167	*Eder & Oberprieler 10298*
**L055-031**	*L. ircutianum* DC. subsp. *ircutianum*	4x	B100627297	DE, Bavaria, Garmisch-Partenkirchen, Laber, 1430 m	47.5833, 11.1167	*Eder & Oberprieler 10298*
**L062-04**	*L. ircutianum* DC. subsp. *ircutianum*	4x	B100416530, B100627403	AT, Vorarlberg, Kleinwalsertal, Baad, 1380 m	47.3000, 10.1000	*Eder, Oberprieler 10304a & Vogt 16222*
**170-02**	*L. ircutianum* subsp. *leucolepis* (Briq. & Cavill.) Vogt & Greuter	4x	B100346645	ME, Herceg Novi, Sutorina, 34 m	42.4742, 18.4731	*Vogt 16724 & Prem-Vogt*
**170-021**	*L. ircutianum* subsp. *leucolepis* (Briq. & Cavill.) Vogt & Greuter	4x	B100346645	ME, Herceg Novi, Sutorina, 34 m	42.4742, 18.4731	*Vogt 16724 & Prem-Vogt*
**174-01**	*L. ircutianum* subsp. *leucolepis* (Briq. & Cavill.) Vogt & Greuter	4x	B100346612, B100346613	ME, Cetinje, Lovćen, Njeguši, 900 m	42.4389, 18.8319	*Vogt 16790 & Prem-Vogt*
**329-02**	*L. ircutianum* subsp. *leucolepis* (Briq. & Cavill.) Vogt & Greuter	4x	B100464093, B100464094	IT, Abruzzo, Pescara, Piano d´Orta, 125 m	42.2447, 13.9659	*Oberprieler 10870*

Sample	Taxon	Ploidy	Voucher specimens	Locality	Coordinates (latitude, longitude)	Collectors
**371-01**	*L. ircutianum* subsp. *leucolepis* (Briq. & Cavill.) Vogt & Greuter	4x	B100486717	HR, Primorje-Gorski Kotar, Vela Učka, 770 m	45.2772, 14.1944	*Vogt 17198 & Prem-Vogt*
**323-03**	*L. meridionale* Legrand	4x	B100464096, B100458572	FR, Occitania, Aveyron, Decanzeville, Firmi, 388 m	44.5514, 2.3044	*Oberprieler 10867*
**323-04**	*L. meridionale* Legrand	4x	B100464096, B100458572	FR, Occitania, Aveyron, Decanzeville, Firmi, 388 m	44.5514, 2.3044	*Oberprieler 10867*
**323-05**	*L. meridionale* Legrand	4x	B100464096, B100458572	FR, Occitania, Aveyron, Decanzeville, Firmi, 388 m	44.5514, 2.3044	*Oberprieler 10867*
**7-02**	*L. pseudosylvaticum* (Vogt) Vogt & Oberpr.	4x	B100413784	PT, Bragança, Izeda – Santulhão, 350 m	41.5582, -6.6754	*Hößl 7 &Hutschenreuther*
**1-05**	*L. pseudosylvaticum* (Vogt) Vogt & Oberpr.	4x	B100413790	ES, Galicia, Lugo, Pedrafita, 1100 m	42.7267, -7.0258	*Hößl 1 & Hutschenreuther*
**3-08**	*L. pseudosylvaticum* (Vogt) Vogt & Oberpr.	4x	B100413788	ES, Galicia, Ourense, Rubiá – O Real, 470 m	42.4634, -6.8950	*Hößl 3 & Hutschenreuther*
