# Supplementary material for: Picks in the Fabric of a Polyploidy Complex: Integrative Species Delimitation in the Tetraploid Leucanthemum Mill. (Compositae, Anthemideae) Representatives
Source: Biology (Basel). 2023 Feb 10;12(2):288. doi: 10.3390/biology12020288 (PMC9953438; doi:10.3390/biology12020288)
Supplement: Supplementary file 1 [file biology-12-00288-s001.zip › ES06.pdf]

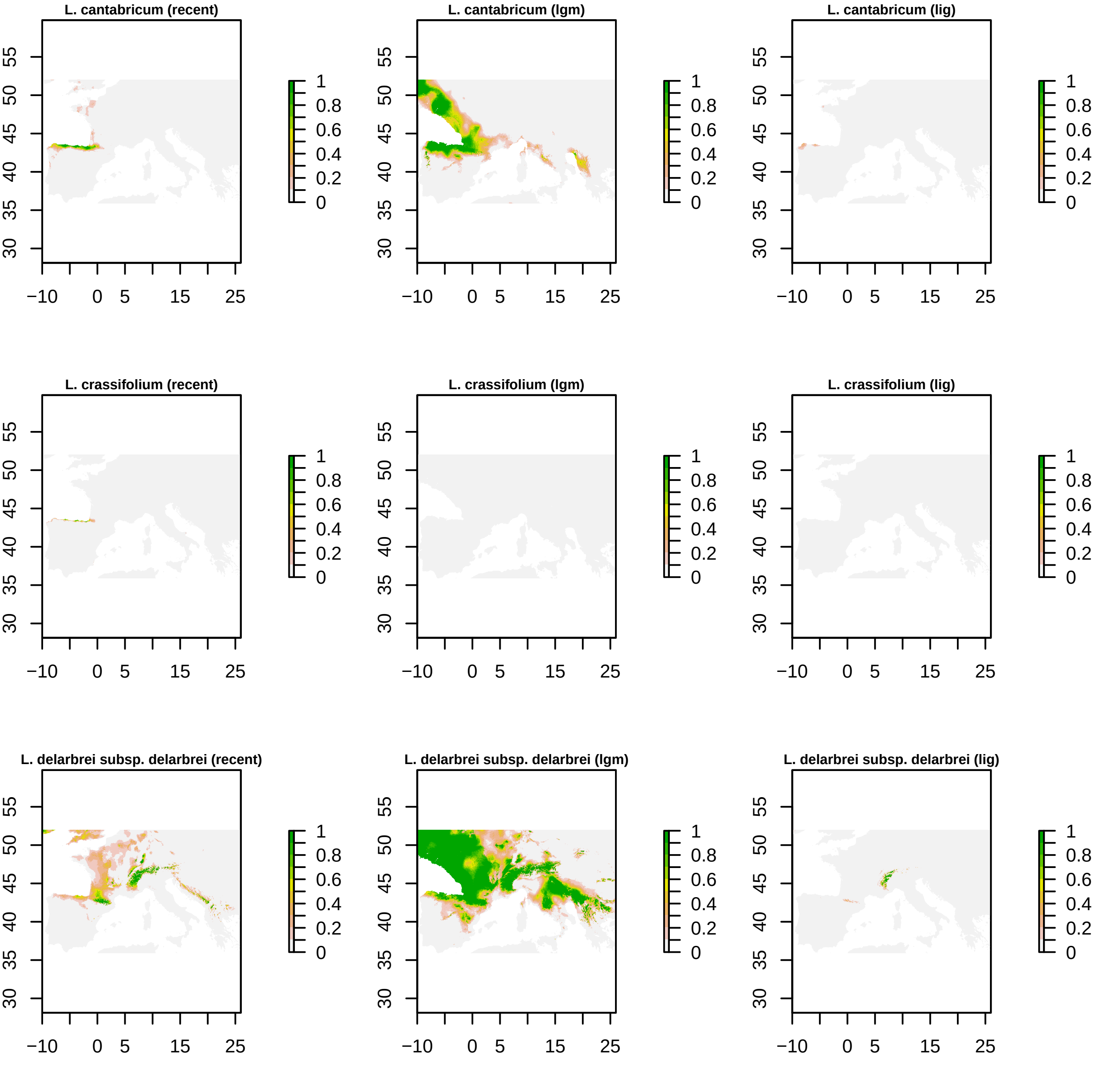
L. cantabricum (recent)
L. cantabricum (lgm)
L. cantabricum (lig)
L. crassifolium (recent)
L. crassifolium (lgm)
L. crassifolium (lig)
L. delarbrei subsp. delarbrei (recent)
L. delarbrei subsp. delarbrei (lgm)
L. delarbrei subsp. delarbrei (lig)
30 35 40 45 50 55
−10 0 5 15 25
0 0.2 0.4 0.6 0.8 1

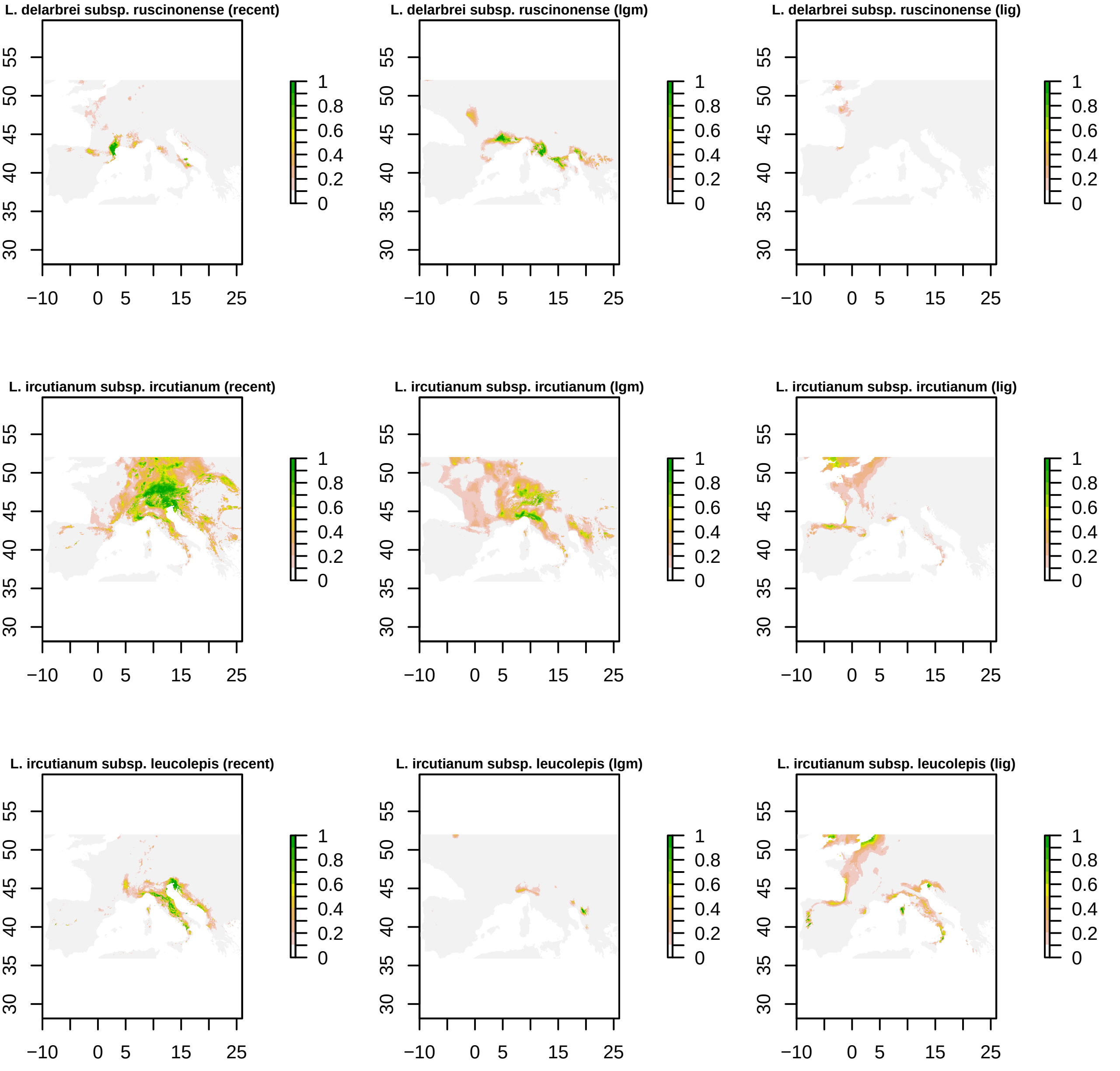
L. delarbrei subsp. ruscinonense (recent)
L. delarbrei subsp. ruscinonense (lgm)
L. delarbrei subsp. ruscinonense (lig)
L. ircutianum subsp. ircutianum (recent)
L. ircutianum subsp. ircutianum (lgm)
L. ircutianum subsp. ircutianum (lig)
L. ircutianum subsp. leucolepis (recent)
L. ircutianum subsp. leucolepis (lgm)
L. ircutianum subsp. leucolepis (lig)
30 35 40 45 50 55
−10 0 5 15 25
0 0.2 0.4 0.6 0.8 1

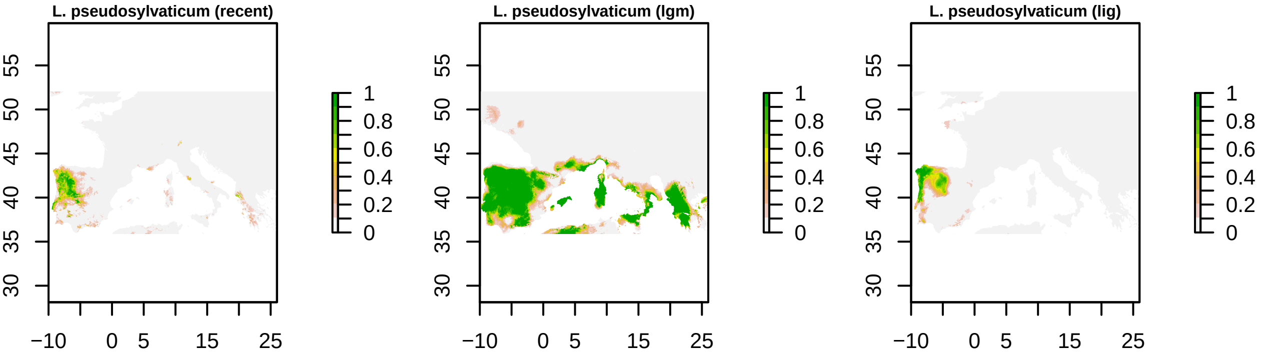
L. pseudosylvaticum (recent)
L. pseudosylvaticum (lgm)
L. pseudosylvaticum (lig)
55
50
45
40
35
30
−10
0
5
15
25
1
0.8
0.6
0.4
0.2
0
