# Supplementary material for: Picks in the Fabric of a Polyploidy Complex: Integrative Species Delimitation in the Tetraploid Leucanthemum Mill. (Compositae, Anthemideae) Representatives
Source: Biology (Basel). 2023 Feb 10;12(2):288. doi: 10.3390/biology12020288 (PMC9953438; doi:10.3390/biology12020288)
Supplement: Supplementary file 1 [file biology-12-00288-s001.zip › ES07.pdf]

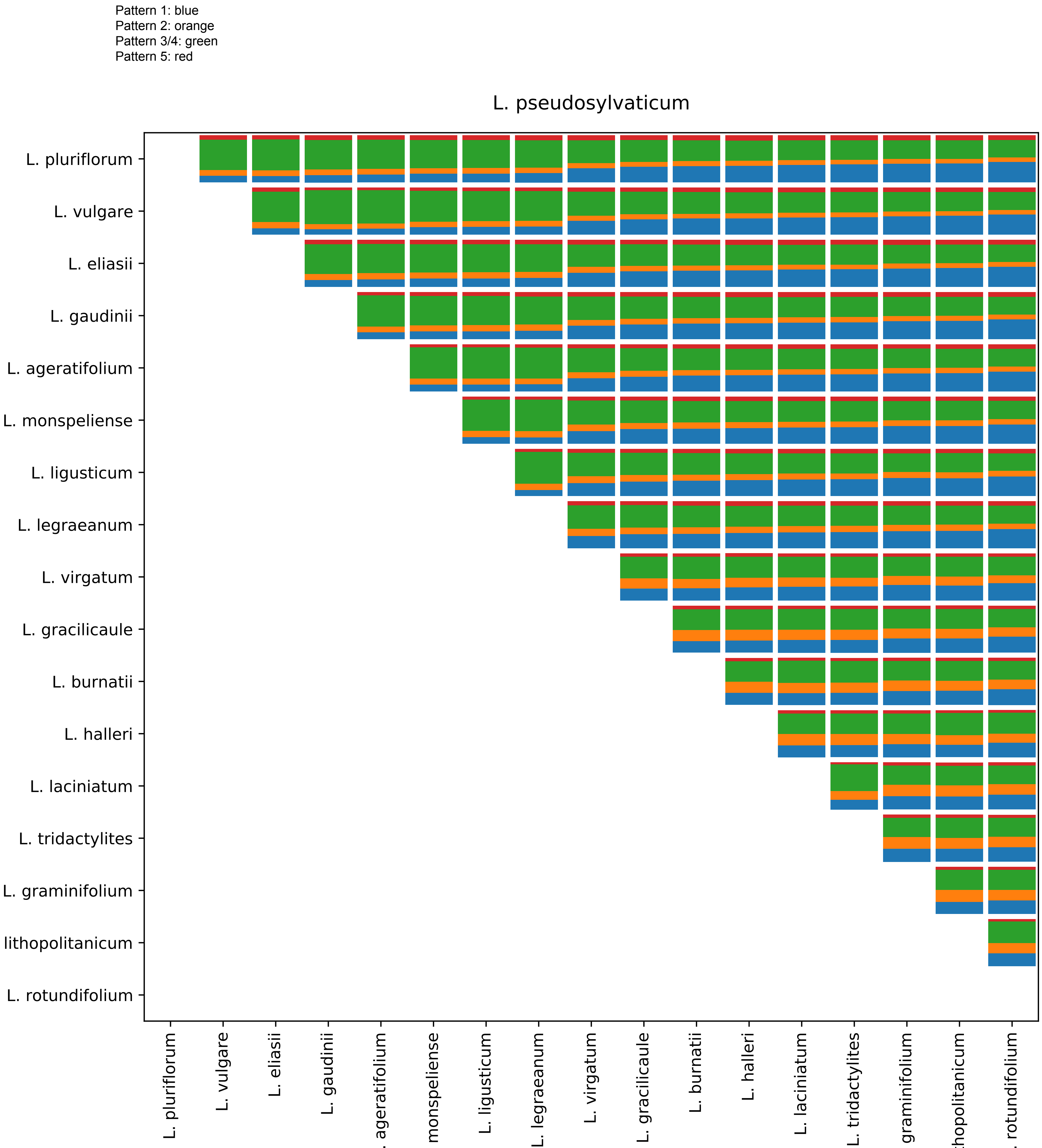

Pattern 1: blue
Pattern 2: orange
Pattern 3/4: green
Pattern 5: red
L. pseudosylvaticum
L. pluriflorum
L. vulgare
L. eliasii
L. gaudinii
L. ageratifolium
L. monspeliense
L. ligusticum
L. legraeanum
L. virgatum
L. gracilicaule
L. burnatii
L. halleri
L. laciniatum
L. tridactylites
L. graminifolium
lithopolitanicum
L. rotundifolium

Pattern 1: blue
Pattern 2: orange
Pattern 3/4: green
Pattern 5: red

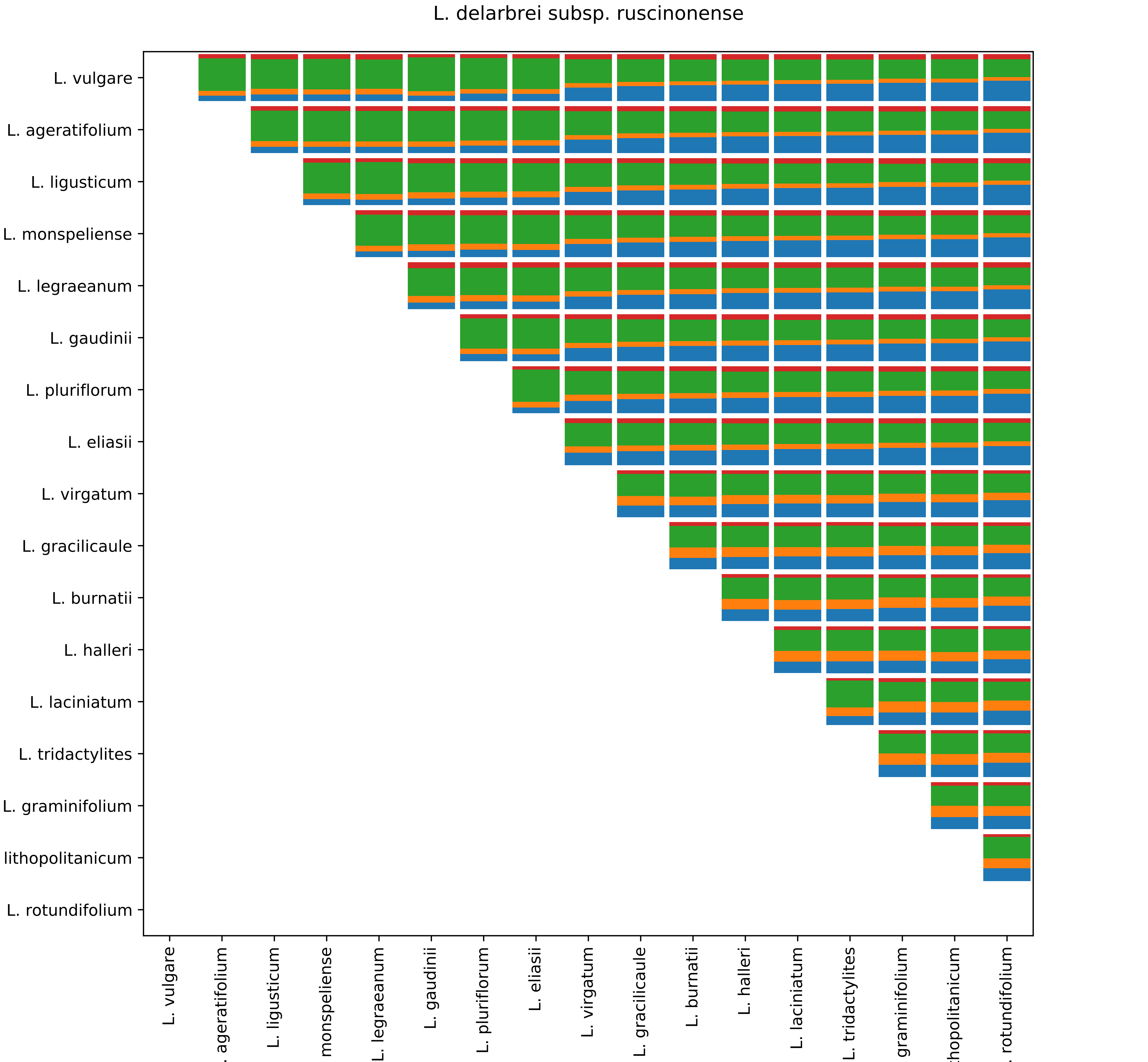

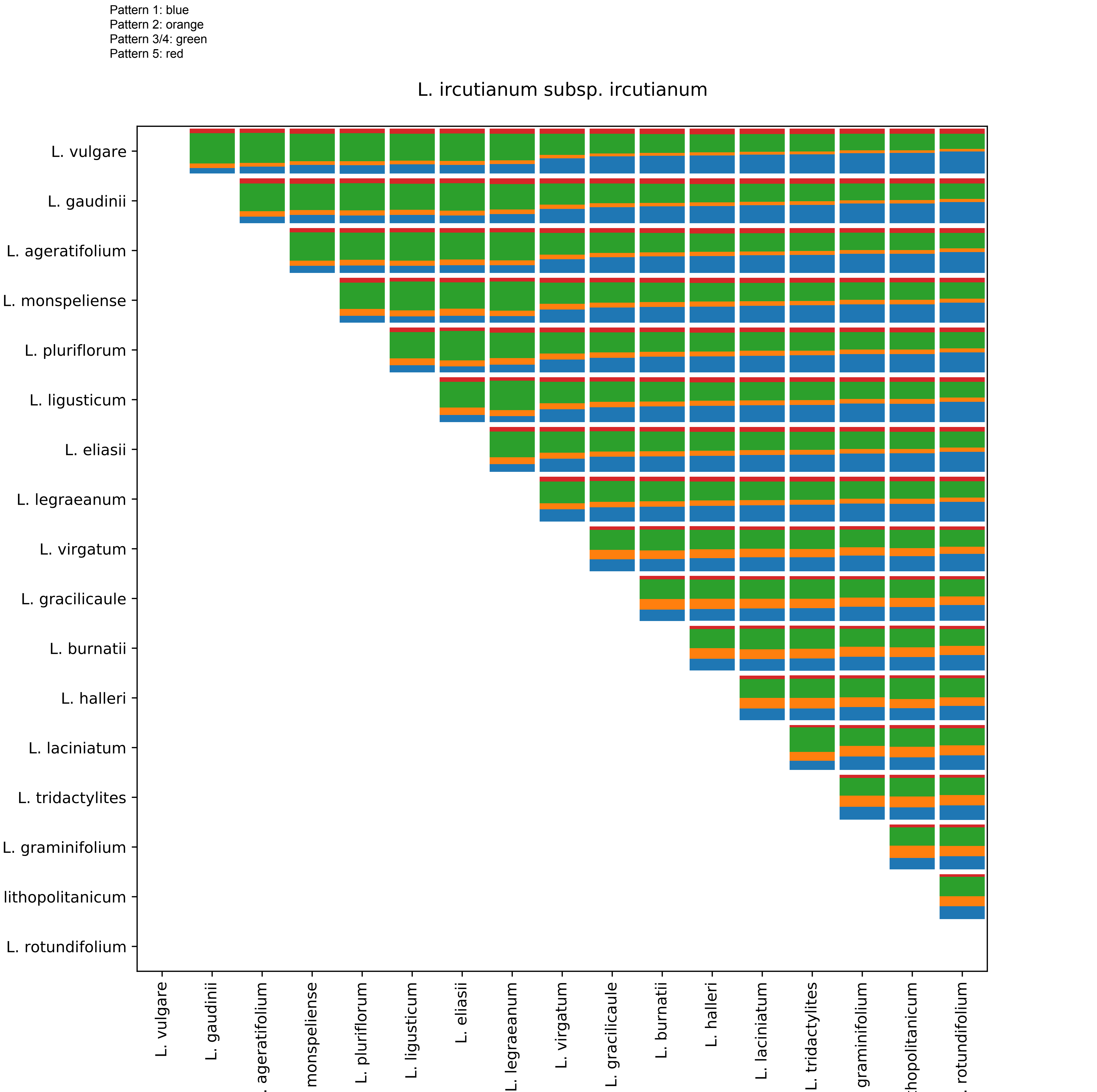

Pattern 1: blue
Pattern 2: orange
Pattern 3/4: green
Pattern 5: red
L. ircutianum subsp. ircutianum
L. vulgare
L. gaudinii
L. ageratifolium
L. monspeliense
L. pluriflorum
L. ligusticum
L. eliasii
L. legraeanum
L. virgatum
L. gracilicaule
L. burnatii
L. halleri
L. laciniatum
L. tridactylites
L. graminifolium
lithopolitanicum
L. rotundifolium
L. vulgare
L. gaudinii
ageratifolium
monspeliense
L. pluriflorum
L. ligusticum
L. eliasii
L. legraeanum
L. virgatum
L. gracilicaule
L. burnatii
L. halleri
L. laciniatum
L. tridactylites
graminifolium
thopolitanicum
rotundifolium

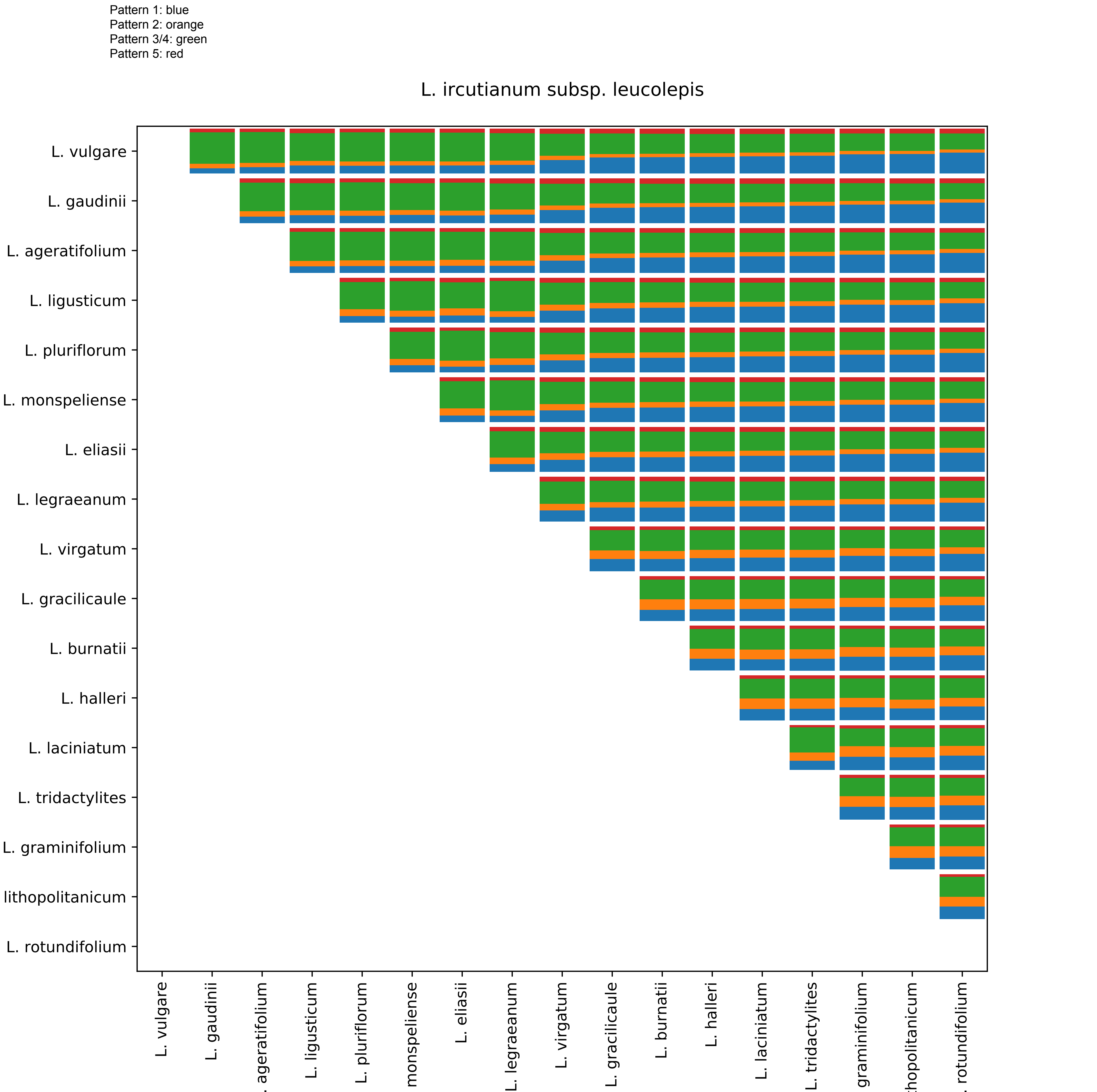
Pattern 1: blue
Pattern 2: orange
Pattern 3/4: green
Pattern 5: red
L. ircutianum subsp. leucolepis
L. vulgare
L. gaudinii
L. ageratifolium
L. ligusticum
L. pluriflorum
L. monspeliense
L. eliasii
L. legraeanum
L. virgatum
L. gracilicaule
L. burnatii
L. halleri
L. laciniatum
L. tridactylites
L. graminifolium
lithopolitanicum
L. rotundifolium

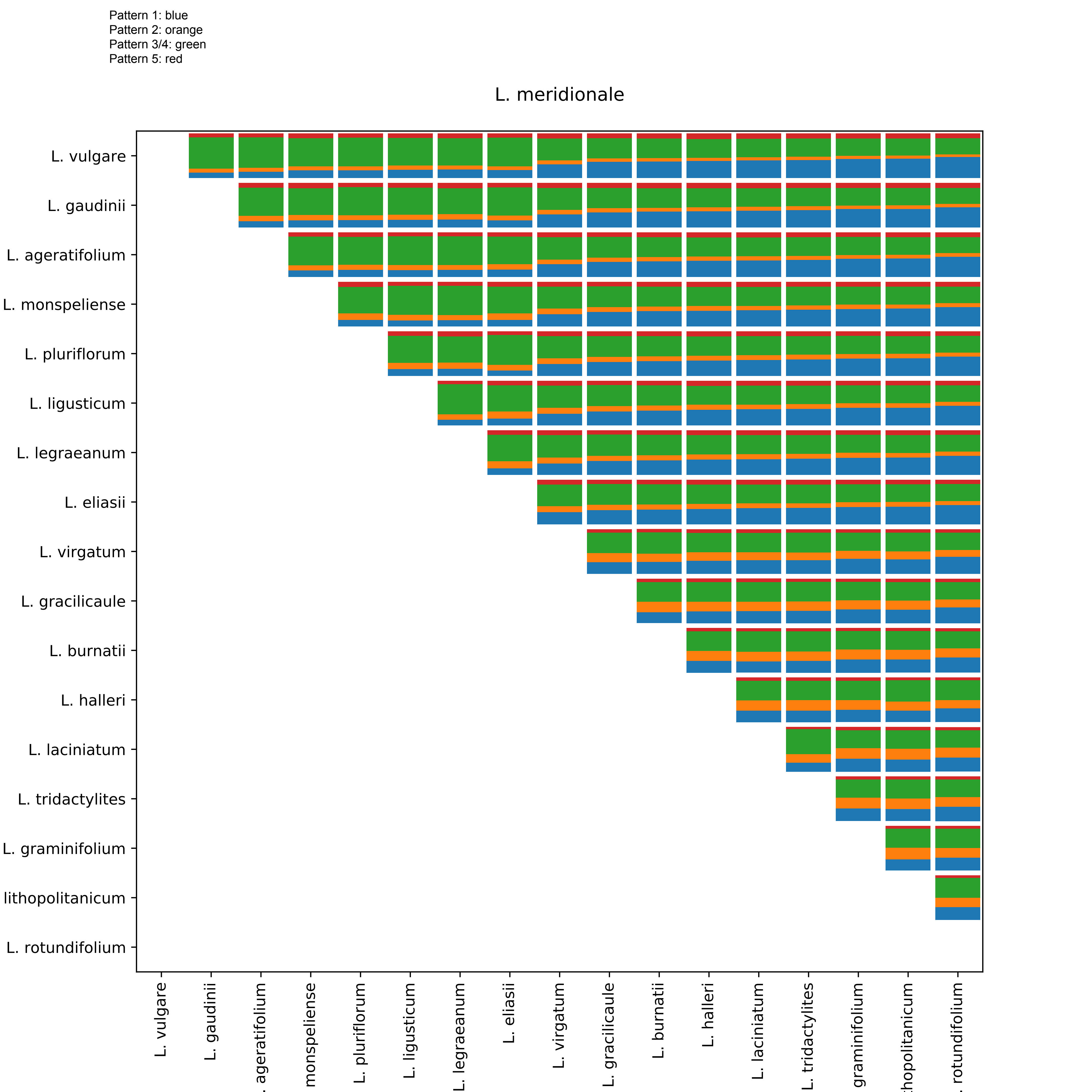
Pattern 1: blue
Pattern 2: orange
Pattern 3/4: green
Pattern 5: red
L. meridionale
L. vulgare
L. gaudinii
L. ageratifolium
L. monspeliense
L. pluriflorum
L. ligusticum
L. legraeanum
L. eliasii
L. virgatum
L. gracilicaule
L. burnatii
L. halleri
L. laciniatum
L. tridactylites
L. graminifolium
lithopolitanicum
L. rotundifolium

Pattern 1: blue
Pattern 2: orange
Pattern 3/4: green
Pattern 5: red

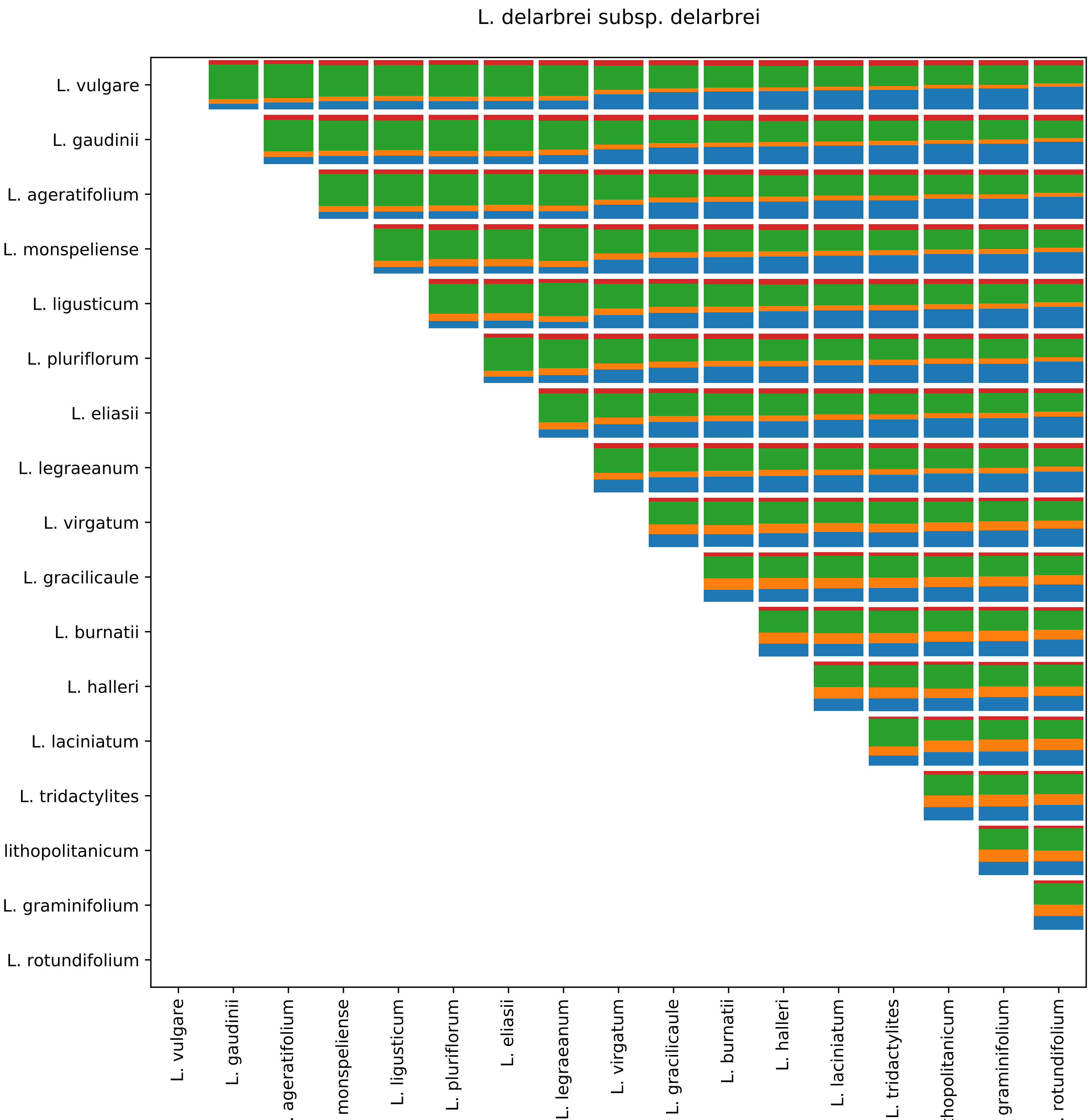

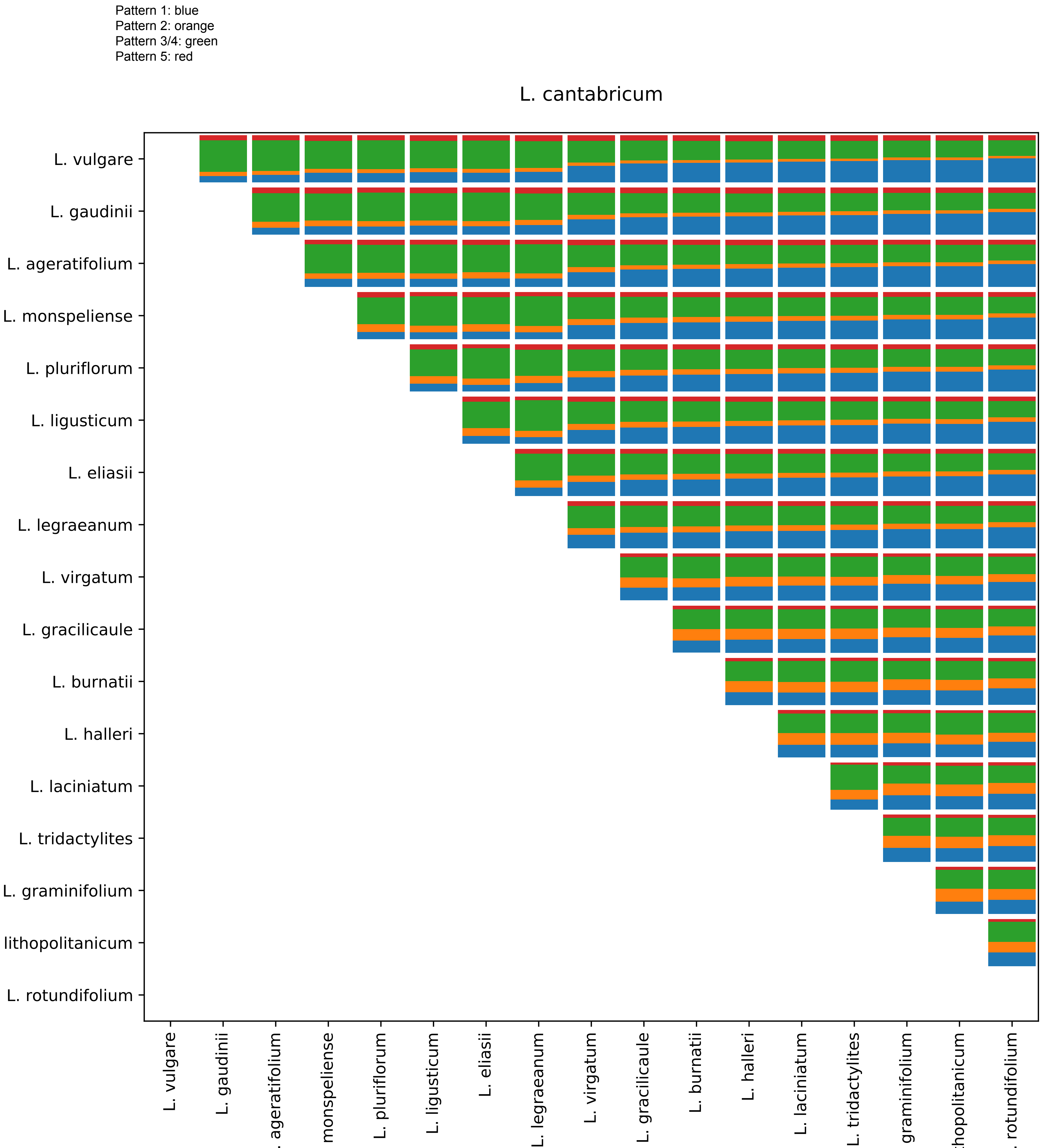

Pattern 1: blue
Pattern 2: orange
Pattern 3/4: green
Pattern 5: red
L. cantabricum
L. vulgare
L. gaudinii
L. ageratifolium
L. monspeliense
L. pluriflorum
L. ligusticum
L. eliasii
L. legraeanum
L. virgatum
L. gracilicaule
L. burnatii
L. halleri
L. laciniatum
L. tridactylites
L. graminifolium
lithopolitanicum
L. rotundifolium

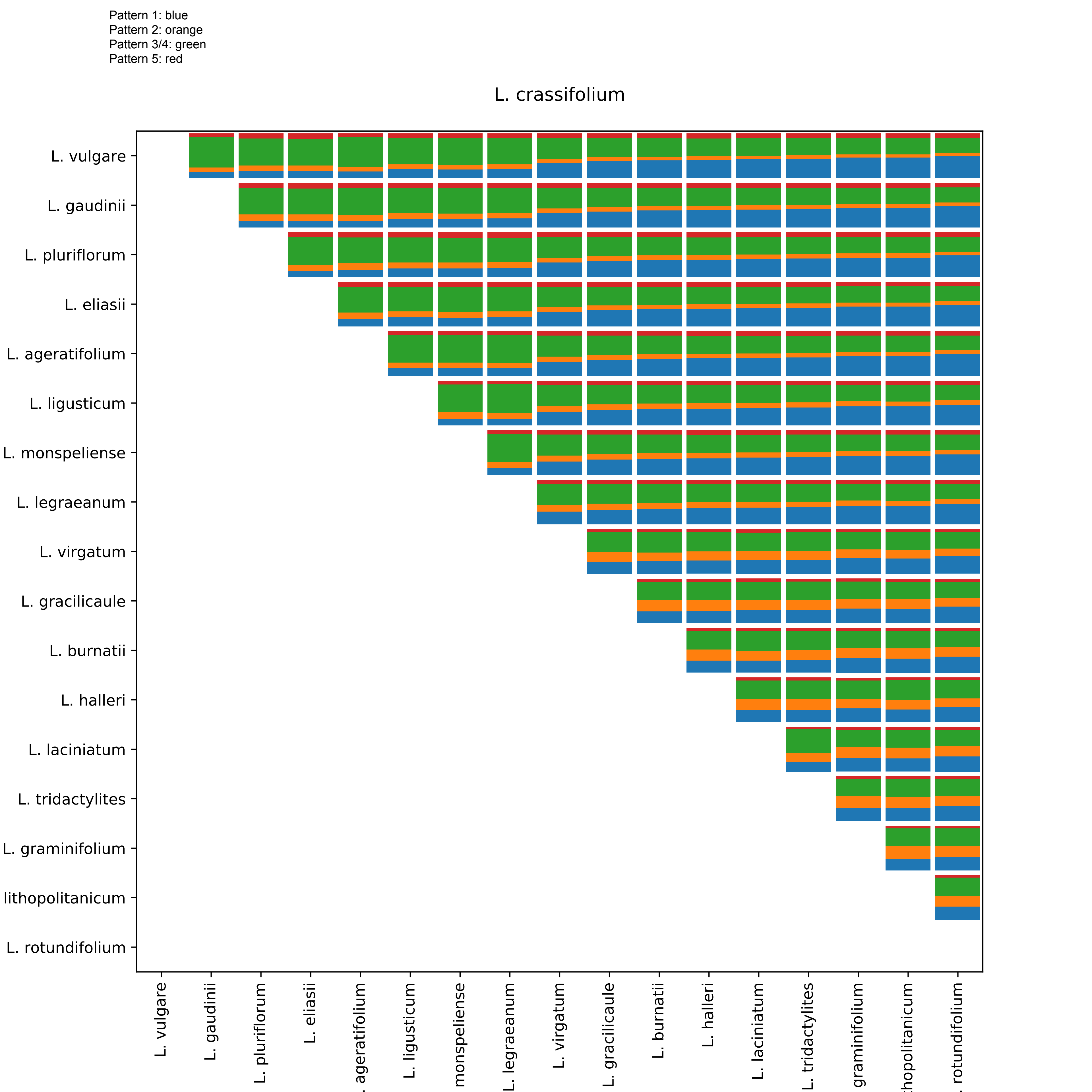
Pattern 1: blue
Pattern 2: orange
Pattern 3/4: green
Pattern 5: red
L. crassifolium
L. vulgare
L. gaudinii
L. pluriflorum
L. eliasii
L. ageratifolium
L. ligusticum
L. monspeliense
L. legraeanum
L. virgatum
L. gracilicaule
L. burnatii
L. halleri
L. laciniatum
L. tridactylites
L. graminifolium
lithopolitanicum
L. rotundifolium
